# UNDERMINING OLDER ADULTS’ AUTONOMY IN THE NAME OF HELP: NEGATIVE AGING ATTITUDES AND PHYSICAL HEALTH OUTCOMES

**DOI:** 10.1093/geroni/igae098.3261

**Published:** 2024-12-31

**Authors:** Zizhuo Chen, Xin Zhang

**Affiliations:** Peking University, beijing, Beijing, China (People’s Republic); Peking University, Beijing, Beijing, China (People’s Republic)

## Abstract

Older adults often face challenges in maintaining autonomy, such as having others make decisions for them and receiving paternalistic help. This research aimed to investigate whether such phenomena, although usually presented in the guise of benevolent help, reflect negative aging attitudes and threaten older adults’ physical health. Study 1 surveyed 89 younger adults on their preference for providing paternalistic (e.g., Developing strict meal plans) versus agentic help (e.g., Offering dietary recommendations with explanations) for different age groups. Results indicated that participants believed older adults (compared with younger adults) were more acceptable to paternalistic help and deemed it more effective than agentic help. Such inclination was positively associated with participants’ demeaning attitudes (i.e., disregarding ones’ advanced psychological needs) towards older adults and fixed essentialist beliefs about aging. Study 2 analyzed a 4-wave, 10-year, nationally representative dataset with 4288 cases to explore the impact of compromised autonomy (having others make decisions for them) on older adults’ physical health. Although cross-lagged model revealed a mutual positive prediction between older adults’ autonomy and their health status, further analysis using instrumental variables confirmed the causal relationship of autonomy on health, rather than the reverse. Moreover, older adults who were less able to make decision for themselves exhibited lower survival probabilities, even after controlling for other variables, including age, health status, ADL, etc. These findings underscored negative attitudes underlying some benevolent helping behaviors that undermined older adults’ autonomy and their detrimental physical outcomes, and shed light on the significance of protecting and promoting older adults’ autonomy.

